# A novel prognostic model based on single-cell RNA sequencing data for hepatocellular carcinoma

**DOI:** 10.1186/s12935-022-02469-2

**Published:** 2022-01-25

**Authors:** Juan Lu, Yanfei Chen, Xiaoqian Zhang, Jing Guo, Kaijin Xu, Lanjuan Li

**Affiliations:** State Key Laboratory for Diagnosis and Treatment of Infectious Diseases, National Clinical Research Center for Infectious Diseases, Collaborative Innovation Center for Diagnosis and Treatment of Infectious Diseases, The First Affiliated Hospital, College of Medicine, Zhejiang University, Shangcheng District, No. 79 Qingchun Road, Hangzhou, 310003 Zhejiang China

**Keywords:** HCC, TIME, Prognosis, scRNA-seq, Risk model

## Abstract

**Background:**

The tumour heterogeneous make-up of immune cell infiltrates is a key factor for the therapy response and prognosis of hepatocellular carcinoma (HCC). However, it is still a major challenge to comprehensively understand the tumour immune microenvironment (TIME) at the genetic and cellular levels.

**Methods:**

HCC single-cell RNA sequencing (scRNA-seq) data were downloaded from the Gene Expression Omnibus (GEO) database, and gene expression data were retrieved from The Cancer Genome Atlas (TCGA) database and International Cancer Genome Consortium (ICGC) database. Cell-type identification by estimating relative subsets of RNA transcripts (CIBERSORT) was performed to evaluate the abundance of immune infiltrating cells. We employed weighted gene coexpression network analysis (WGCNA) to construct a gene coexpression network. Univariate Cox and least absolute shrinkage and selection operator (LASSO) analyses were further used to construct a risk model. Moreover, the expression levels of model genes were assessed by qPCR.

**Results:**

We defined 25 cell clusters based on the scRNA-seq dataset (GSE149614), and the clusters were labelled as various cell types by marker genes. Then, we constructed a weighted coexpression network and identified a total of 6 modules, among which the brown module was most highly correlated with tumours. Moreover, we found that the brown module was most closely related to monocytes (cluster 21). Through univariate Cox and LASSO analyses, we constructed a 3-gene risk model (RiskScore = 0.257*Expression _CSTB_ + 0.263* Expression _TALDO1_ + 0.313* Expression _CLTA_). This risk model showed excellent predictive efficacy for prognosis in the TCGA-LIHC and ICGC cohorts. Additionally, patients with high risk scores were found to be less likely to benefit from immunotherapy.

**Conclusions:**

We developed a 3-gene signature (including CLTA, TALDO1 and CSTB) based on the heterogeneity of the TIME to predict the survival outcome and immunotherapy response.

**Supplementary Information:**

The online version contains supplementary material available at 10.1186/s12935-022-02469-2.

## Background

Hepatocellular carcinoma (HCC) is the most common primary liver cancer and accounts for 75–85% of cases. HCC was also the sixth most commonly diagnosed cancer and the fourth leading cause of cancer-related deaths globally [1] in 2018. Only hepatic resection and liver transplantation are considered potentially curative approaches for treating HCC. However, most patients are diagnosed at a late stage, and the treatment rate for early-stage patients is disappointingly low [2]. It is well known that the tumour immune microenvironment (TIME) plays an essential role in tumorigenesis, tumour development, and treatment outcome [3–5]. In recent years, immunotherapy has emerged as a promising strategy for cancer treatment, while only a few HCC patients showed response to immune treatment. Therefore, systematic analysis of the function of various types of intratumour immune cells might contribute to the development of novel biomarkers for prognosis and therapeutic effectiveness for patients with HCC.

With the rapid development of next-generation sequencing technologies, an increasing number of studies have examined gene expression in HCC based on RNA sequencing (RNA-seq). However, RNA-seq is typically performed in “bulk”, with data representing the average gene expression patterns of a large number of cells [6]. Notably, single-cell RNA sequencing (scRNA-seq) is a novel sequencing technology that provides relevant information for the characterization of single immune cells or tumour cells [7]. scRNA-seq highlights intratumour heterogeneity and distinct subpopulations, and it is possible to enumerate and quantify immune infiltration in tumour tissues [8, 9]. Importantly, the heterogeneous make-up of immune cell infiltrates is a key factor for therapy response and prognosis in HCC and other tumour types [10–14]. Unfortunately, scRNA-seq is relatively expensive, so only a limited number of sample datasets were available. However, the information from scRNA-seq can be very meaningful for exploring the characteristics of each cell subpopulation from bulk samples and the interaction of each cell in the TIME [15–17].

In the present study, distinct cell subpopulations between tumour tissues and normal control tissues were identified from HCC scRNA-seq datasets in the Gene Expression Omnibus (GEO) database. The weighted gene coexpression network analysis (WGCNA) algorithm was used to explore the coexpression network and key modules most closely related to tumours based on the Cancer Genome Atlas (TCGA) expression profile data of tumour samples and normal samples. Based on the integration of scRNA-seq and bulk RNA-seq data, we screened the key genes related to immune cell subsets in HCC. Next, we employed univariate Cox and least absolute shrinkage and selection operator (LASSO) Cox regression to construct a risk model, which was demonstrated to have great potential as a biomarker for prognosis and to have excellent predicted immunotherapeutic efficacy for patients with HCC.

## Methods

### Data source and preprocessing

The HCC scRNA-seq dataset GSE149614 was downloaded from the GEO database and included 10 primary tumour (PT) patients, 2 portal vein tumour thrombus (PVTT) patients, 1 metastatic lymph node (MLN) patient and 8 normal liver tissue (NLT) patients. The original data contained a total of 25,479 genes and 71,915 cells. The percentage of mitochondria and rRNA was calculated through the PercentageFeatureSet function, and the genes expressed by each cell were greater than 500 and less than 8000, the selection criteria was showed in Additional file 2: Figure S1. The mitochondrial content was less than 30%. In addition, the number of UMIs in each cell was at least 500. After filtration, there were 71,139 cells.

Public clinical data and gene expression information were retrieved from the TCGA database (https://portal.gdc.cancer.gov/) and International Cancer Genome Consortium (ICGC) database (https://xena.ucsc.edu/). In total, 366 samples in the TCGA-LIHC cohort and 232 samples in the ICGC-JP cohort were used for further analysis.

### scRNA-seq data clustering dimension reduction

First, we normalize the merged data through log-normalization and find the first 2000 highly variable genes through the FindVariableFeatures function (identify variable features based on the variance stabilization transformation (“vst”)). At the same time, all genes were scaled using the ScaleData function, and RunPCA function was used to reduce the dimension of PCA for the first 2000 highly variable genes screened above. We choose dim = 50 and clustered the cells through the “FindNeighbors” and “FindClusters” functions (resolution = 0.1) to find the cell clusters. Next, we selected the top 50 principal components to further reduce dimensionality using the UMAP method. UMAP is a method of data dimensionality reduction, which assumes that the available data samples are uniformly distributed in the topological space (Manifold), and these limited data samples can be approximated (Approximation) and mapped (Projection) to a low-dimensional space. To put it simply, the UMAP algorithm is considered to be a principle similar to t-SNE, which is an algorithm that maps the high-dimensional probability distribution to a low-dimensional space, so as to achieve the effect of dimensionality reduction. Mainly based on the theory of manifold theory and topology algorithm, the dimensionality of high-dimensional data is reduced to form the input features of other classification models. Finally, we used the FindAllMarkers function to screen the marker genes of 25 subgroups with logfc = 0.5 (differential multiples) and Minpct = 0.35 (the expression ratio of the least differential genes). Finally, we used the corrected p < 0.05 to screen the marker gene.

### Cell-type identification by estimating relative subsets of RNA transcripts (CIBERSORT)

CIBERSORT is a method based on the input matrix of a gene expression file to accurately estimate the relative proportions of various cell subsets in tissues [18, 19]. Here, we used CIBERSORT analysis to compare differences in various immune cells in distinct groups. Spearman correlation analysis was performed to explore the association between the risk score and infiltrating immune cells. The “ggplot2” package was used to visualize the differences in abundance in immune cells and the results of the correlation analysis.

### Least absolute shrinkage and selection operator (LASSO)

To construct the prognostic model, a univariate Cox regression model identified the genes that were significantly correlated with survival outcome. Moreover, LASSO analysis was employed to select reliable predictors [20]. The risk score of each patient in the TCGA database and ICGC database was assessed using the formula risk score = Σ coefficient_mRNAn_ * expression level _mRNAn_. Then, the correlation between the risk score and prognosis of patients was further analysed.

### Weighted gene co-expression network analysis (WGCNA)

WGCNA is an R software package that is used for weighted correlation network analysis, including for module identification, network generation, gene screening, calculation of properties, and data visualization [21]. Here, we used cibersort's algorithm to evaluate the score of each sample of TCGA bulk RNA-seq with respect to each cell subgroup. Each sample itself is a geometric body of multiple cell types. We performed WGCNA analysis on this similarity score to screen the gene modules with the highest correlation with a certain subgroup. [22] Highly similar modules were identified by cluster analysis, and the association between each module and intratumour cell subgroup abundance was assessed.

### Tumour immune dysfunction and exclusion (TIDE)

To predict the immune checkpoint blockade response, Jiang et al. developed the TIDE method, which was used to simulate the mechanisms of tumour immune evasion (including T cell dysfunction and T cell exclusion) [23, 24]. The software is freely available online at http://tide.dfci.harvard.edu. In this study, we employed TIDE to evaluate the response to immunotherapy in patients. A higher TIDE score indicates a higher possibility of immune escape and poor response to immunotherapy for patients with HCC.

### Cell culture and quantitative real-time PCR (qRT-PCR)

The HCC cell line SK-Hep-1 and healthy human liver cell line L02 were obtained from the Chinese Academy of Sciences. The cells were maintained in Dulbecco’s modified Eagle’s medium supplemented in 10% foetal bovine serum (Wisent, Ottawa, ON, Canada) and 1% penicillin in humid conditions at 37 °C with a 5% CO2 atmosphere. The RNA of the cell lines L02 and SK-Hep-1 was extracted by using TRIzol reagent (Invitrogen), and the RevertAid First-Strand cDNA Synthesis Kit (Thermo Fisher Scientific, Inc.) was used to synthesize cDNA. qRT-PCR analysis was performed using SYBR Green (Takara). The primer sequences are listed in Additional file 1: Table S1.

### Statistical analysis

Prism 7.0 (GraphPad software, CA, USA) and R version 3.5.2 were used for statistical analysis. Kaplan–Meier survival curves were used for survival analysis by the survminer R package version 2.43–3. Student’s t-test was carried out to analyse the significant differences among distinct groups. The glmnet R package was used for LASSO Cox regression analysis. A P-value < 0.05 indicated statistical significance (*P < 0.05; **P < 0.01; ***P < 0.001; ****P < 0.0001).

## Results

### Definition of clusters and dimensionality reduction for visual representation of the cells

**Fig. 1:**
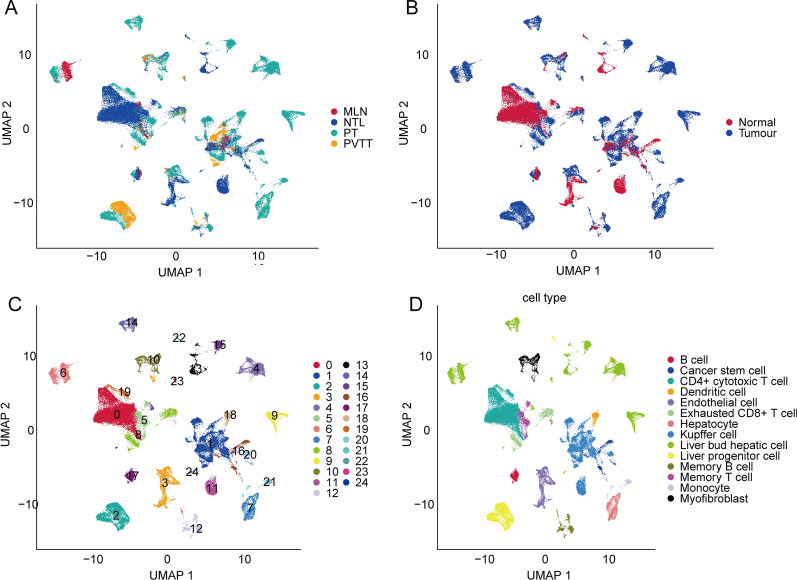
Overview of single cells from tumour samples and normal samples. **a** Umap of four different types of samples. **b** The sample types of the cells. **c** Umap of the 25 cell clusters. **d** The cell types were identified by marker genes. Abbreviations: MLN, metastatic lymph node; MTL, metastatic lymph node; PT, primary tumour; PVTT, portal vein tumour thrombus

The overall workflow was showed in Additional file 3: Figure S2. We performed the “ScaleData” function to scale all genes extracted from the scRNA-seq dataset GSE149614 and performed PCA dimensionality reduction to find anchor points. Finally, 25 clusters were found (Additional file 3: Figure S2a and b). We screened the cell markers of the 25 clusters by the “FindAllMarkers” function (logfc = 0.5, Minpct −0.35), and the top 5 genes with the most prominent contributions are shown in Additional file 4: Figure S3c. An overview of the single cells from four types of samples is shown in Fig. 1a. Cells originating from tumour tissues and normal control tissues are shown in Fig. 1b. All the cells were classified into 25 clusters (Fig. 1c). These identified clusters were labelled as various cell types by marker genes (Fig. 1d). Moreover, we downloaded the human cell marker gene from CellMarker (http://biocc.hrbmu.edu.cn/CellMarker/) and defined 25 cell subsets using the clusterProfiler package enricher function. Information on the cell subsets is shown in Table 1.

**Table 1:** The information of 25 cells types

Cluster	Cell type
0	CD4 + cytotoxic T cell
1	Kupffer cell
2	Liver progenitor cell
3	Endothelial cell
4	Liver bud hepatic cell
5	5 Memory T cell
6	Liver bud hepatic cell
7	Hepatocyte
8	Exhausted CD8 + T cell
9	Liver bud hepatic cell
10	Myofibroblast
11	Kupffer cell
12	Memory B cell
13	Liver bud hepatic cell
14	Liver bud hepatic cell
15	Liver bud hepatic cell
16	Cancer stem cell
17	B cell
18	Dendritic cell
19	CD4 + cytotoxic T cell
20	Kupffer cell
21	Monocyte
22	Liver progenitor cell
23	Liver bud hepatic cell
24	Dendritic cell

### Definition of cell subgroups

**Fig. 2:**
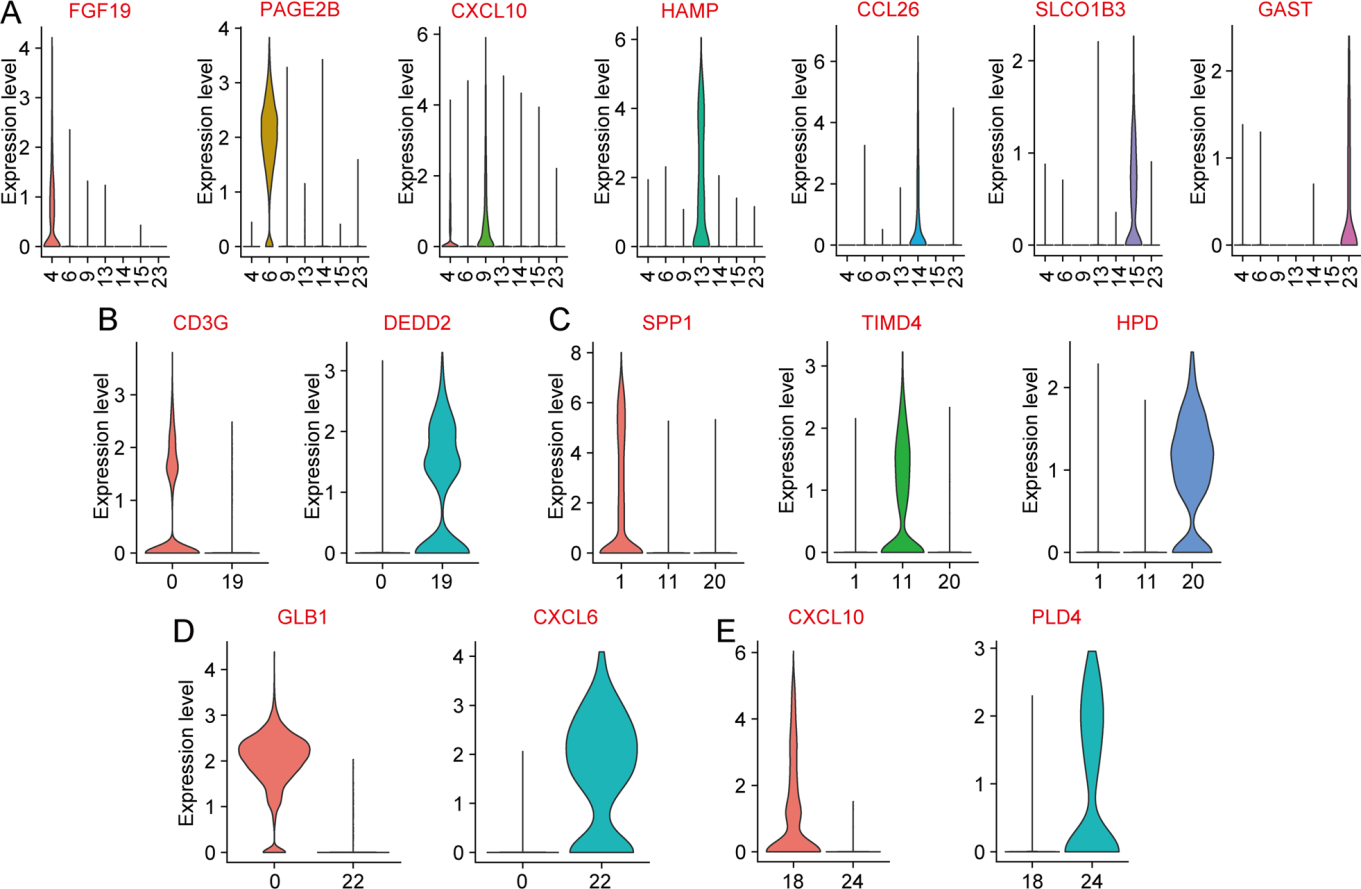
Violin diagram of characteristic gene expression of 5 subgroups. The specifically expressed marker genes of liver bud hepatic cells (**a**), CD4 + cytotoxic T cells (**b**), Kupffer cells (**c**), liver progenitor cells (**d**), and dendritic cells (**e**)

Notably, we found multiple subgroups of 5 cell types, including liver bud hepatic cells, CD4 + cytotoxic T cells, dendritic cells, Kupffer cells, and liver progenitor cells. For liver bud hepatic cell cells, the C4 subgroup specifically expressed the FGF19 gene, cluster 6 (C6) specifically expressed the PAGE2B gene, C9 specifically expressed the CXCL10 gene, C13 specifically expressed the HAMP gene, C14 specifically expressed the CCL26 gene, C15 specifically expressed the SLCO1B3 gene, and the C23 subgroup specifically expressed the GAST gene (Fig. 2a). The specifically expressed marker genes of CD4 + cytotoxic T cells (Fig. 2b), Kupffer cells (Fig. 2c), liver progenitor cells (Fig. 2d), and dendritic cells (Fig. 2e) were also identified. These results indicated that the specially expressed marker genes might be used to identify the subgroups of cells in future studies.

### Identification of coexpression modules in HCC

**Fig. 3:**
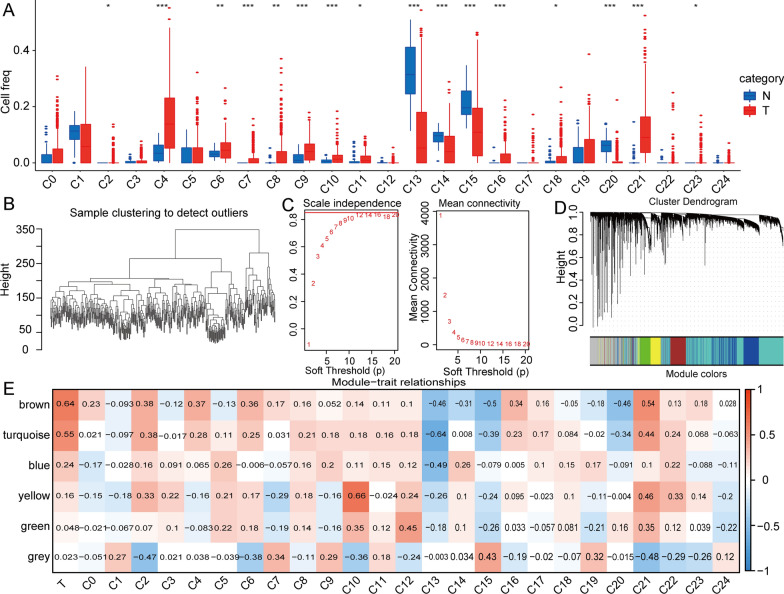
Identification of co-expression modules in HCC. **a** The abundance of the 25 identified clusters in tumour tissues and paracarcinoma tissues from the TCGA database by the CIBERSORT method. **b** Sample clustering to detect outliers. **c** The scale-free fit index for soft-thresholding powers. **d** Constructing a gene dendrogram based on different metrics. **e** Correlation analysis between 6 modules and each cell subset. Abbreviations: HCC, hepatocellular carcinoma; TCGA, The Cancer Genome Atlas; CIBERSORT, Cell-type identification by estimating relative subsets of RNA transcripts

To explore the characteristics of the TIME in HCC, we calculated the abundance of the 25 identified cell clusters in tumour tissues and paracarcinoma tissues from the TCGA database by the CIBERSORT method. We found that the abundances of 16 cell subgroups were different between tumour tissues and normal tissues, including liver progenitor cells (C2), liver bud hepatic cells (C4), liver bud hepatic cells (C6), hepatocytes (C7), exhausted CD8 + T cells (C8), liver bud hepatic cells (C9), myofibroblasts (C10), Kupffer cells (C11), liver bud hepatic cells (C13), liver bud hepatic cells (C14), liver bud hepatic cells (C15), cancer stem cells (C16), dendritic cells (C18), Kupffer cells (C20), monocytes (C21), and liver bud hepatic cells (C23) (Fig. 3a).

To further analyse the correlation between gene expression patterns and distinct cell subgroups in HCC, we used the WGCNA method to construct key modules based on the expression profile data of 371 tumour tissues and 50 normal tissues of the TCGA-LIHC cohort. The results of hierarchical clustering analysis of all samples are shown in Fig. 3b. We utilized Pearson’s correlation coefficient to calculate the distance between each gene and used WGCNA to construct a scale-free network (Fig. 3c). Then, we utilized the average-linkage hierarchical clustering method to cluster genes, and a total of 6 modules were obtained, among which the grey module was a gene set that would not be clustered in other modules (Fig. 3d). We further analysed the correlation between each module and the abundances of cell subgroups (Fig. 3e). We found that cancer was most closely related to the brown module, which was most closely related to monocytes (C21). Additionally, to explore the functional annotation of the genes in the brown module, we performed KEGG and GO enrichment analyses. For the GO functional annotations of genes, 145 terms were enriched in biological process (BP) (FDR < 0.05). The top 10 annotation results are shown in Additional file 4: Figure S3a. The top 10 terms enriched in molecular function (MF) are shown in Additional file 5: Figure S4b. The top 10 annotation results are shown in Additional file 4: Figure S3c. The results of KEGG pathway enrichment analysis of the top 10 annotations are shown in Additional file 4: Figure S3d.

### Construction of the prognostic model based on the key genes

**Fig. 4:**
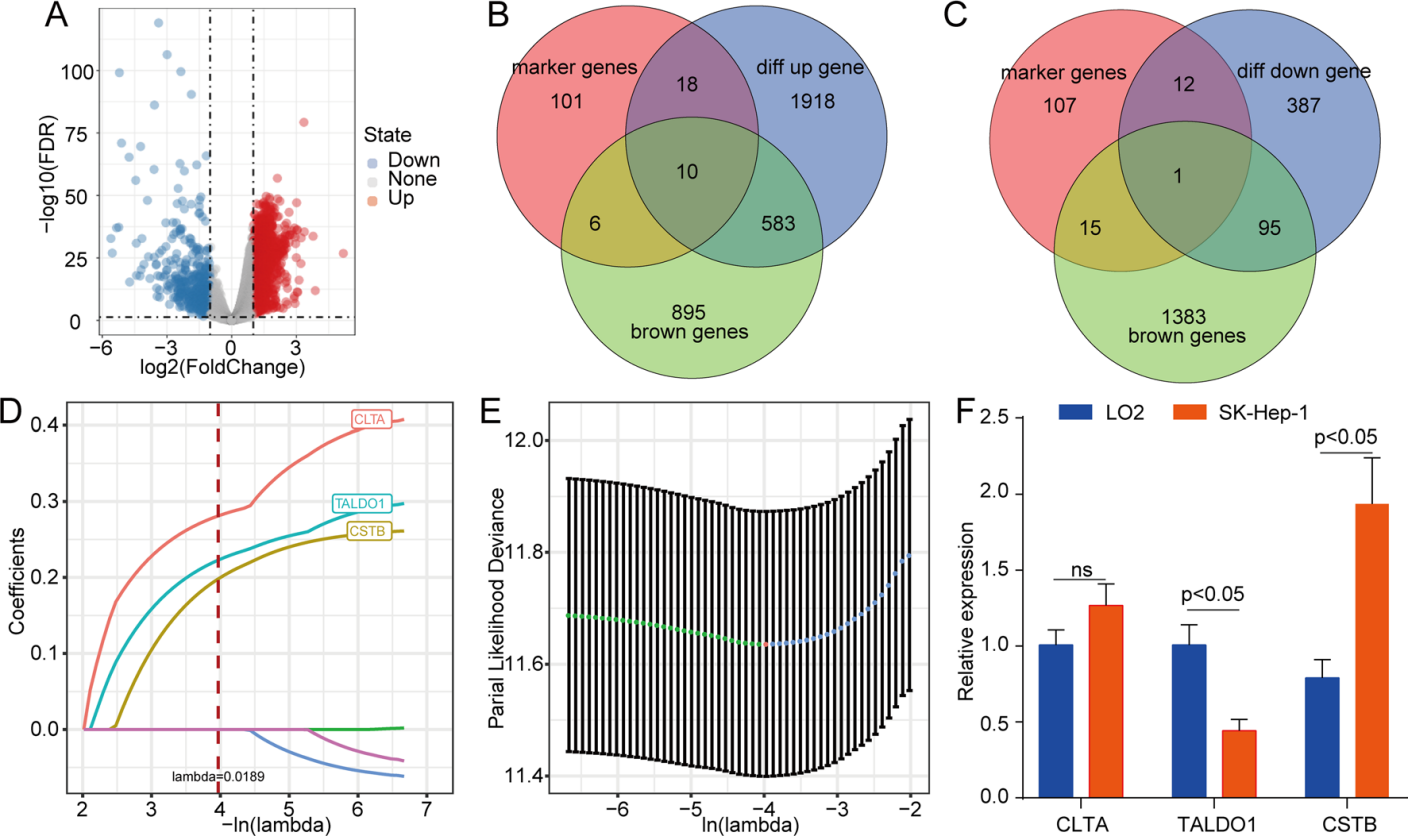
Construction of the risk model based on the key genes. a The volcano map of difference analysis in the TCGA-LIHC cohort. **b** Venn diagram of tumorigenesis-related upregulated genes, monocyte (C21) marker genes and brown module genes. **c** Venn diagram of tumorigenesis-related downregulated genes, monocyte (C21) marker genes and brown module genes. **d** LASSO coefficient profile plots of each independent variable. **e** The partial likelihood deviance for the LASSO Cox regression analysis. **f** The expression of CLTA, TALDO1, and CSTB in the HCC cell line SK-hep-1 and the normal live cell line LO2, as determined by qRT-PCR. Abbreviations: TCGA-LIHC, The Cancer Genome Atlas Liver Hepatocellular Carcinoma; HCC, hepatocellular carcinoma; qRT-PCR, quantitative real time polymerase chain reaction

To screen key genes related to tumorigenesis, we performed differential expression analysis on gene expression data from the TCGA database using the R package limma. A total of 3864 differentially expressed genes (DEGs) were identified, of which 2529 genes were upregulated and 495 were downregulated (Fig. 4a). Through overlap analysis of the upregulated genes, the brown module gene and the monocyte (C21) marker gene, we found a total of 10 genes in the brown module that were upregulated genes and belonged to the C21 marker genes (Fig. 4b) and 1 gene in the brown module that was a downregulated gene and belonged to C21 marker genes (Fig. 4c). Then, a univariate Cox regression model identified 7 genes in the TCGA-LIHC cohort that were significantly correlated with overall survival. LASSO Cox regression analysis was used to further reduce the number of candidate genes. The change trajectory of each gene is shown in Fig. 4d. At the same time, before we perform these analyses, we have performed corresponding preprocessing on the TCGA data, adding 1 to the value of the original expression profile, and using the logarithm of 2 as the logarithm, and then filtering the matrix with the sample variance greater than 0.5. Three genes, CLTA, TALDO1 and CSTB, were identified and used to generate a risk model (Fig. 4e). The 3-gene model formula was as follows: RiskScore = 0.257 * Expression _CSTB_ + 0.263 * Expression _TALDO1_ + 0.313 * Expression _CLTA_. We further assessed the expression of CLTA, TALDO1, and CSTB in the HCC cell line SK-Hep-1 and the healthy live cell line LO2 by qRT-PCR. The results showed that the three genes were all upregulated in the HCC cell line (Fig. 4f).

### Evaluation of the predictive efficiency of the risk model for prognosis in TCGA-LIHC and ICGC cohorts.

**Fig. 5:**
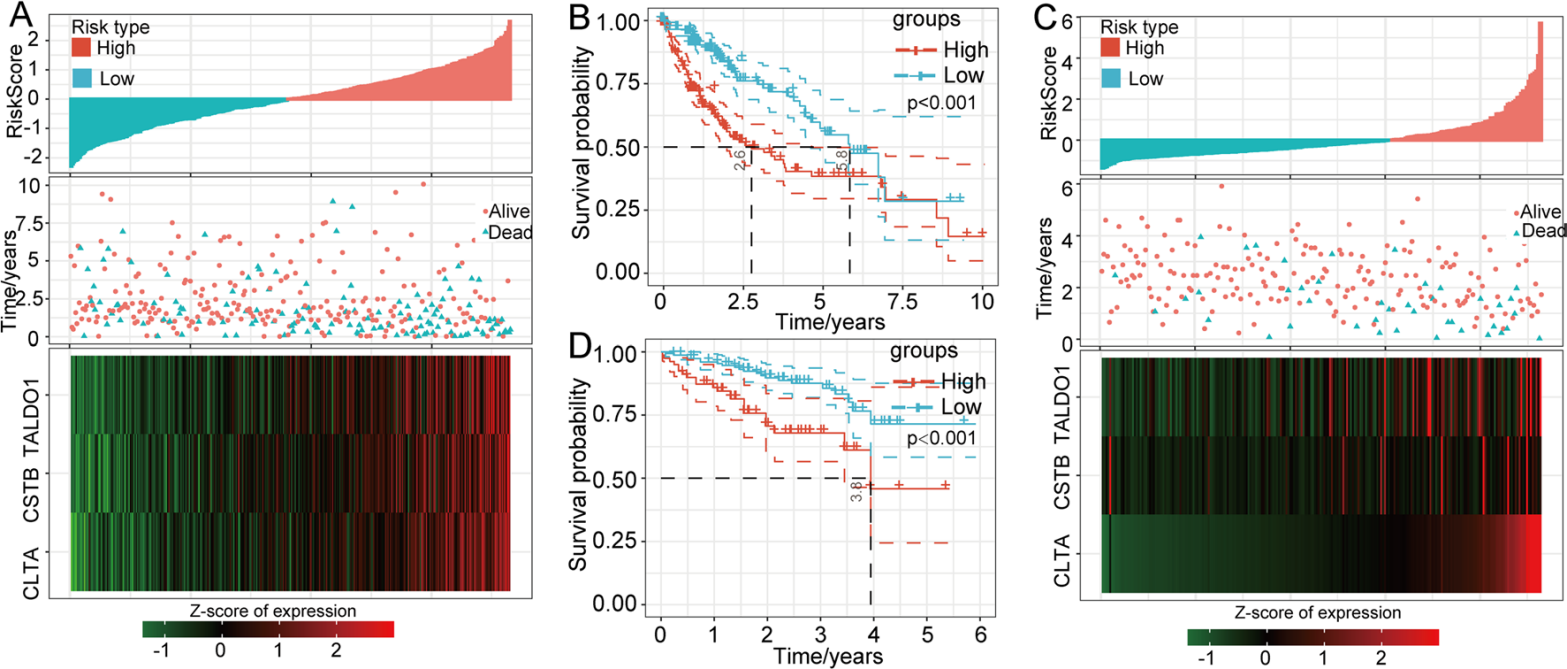
Evaluation and validation of the predictive efficacy of the prognostic model. **a** The risk score distribution, patient status and mRNA expression heatmap for the TCGA-LIHC cohort. **b** Kaplan–Meier curves of the 3-gene model for the TCGA-LIHC cohort. **c** The risk score distribution, patient status and mRNA expression heatmap for the ICGC cohort. **d** Kaplan–Meier curves of the 3-gene model for the ICGC cohort. Abbreviations: TCGA-LIHC, The Cancer Genome Atlas Liver Hepatocellular Carcinoma; ICGC, International Cancer Genome Consortium

After construction of the 3-gene model, we calculated the risk scores based on the model for each patient in the TCGA-LIHC cohort and plotted the risk score distribution of the patients. We discovered that patients with a high risk score (n = 185) had a markedly higher risk of death than those with a low risk score (n = 180) (Fig. 5a). The results of survival analysis also showed that high-score patients had a poorer prognosis than low-score patients (Fig. 5b). To further verify the predictive performance of our model, we tested this model in the ICGC database (Fig. 5c). Similarly, the high-score group presented a significantly shorter overall survival time than the low-score group (Fig. 5d). All this evidence indicated that we had constructed an excellent risk model for prognosis.

### Association between risk score and infiltrating immune cells in HCC

**Fig. 6:**
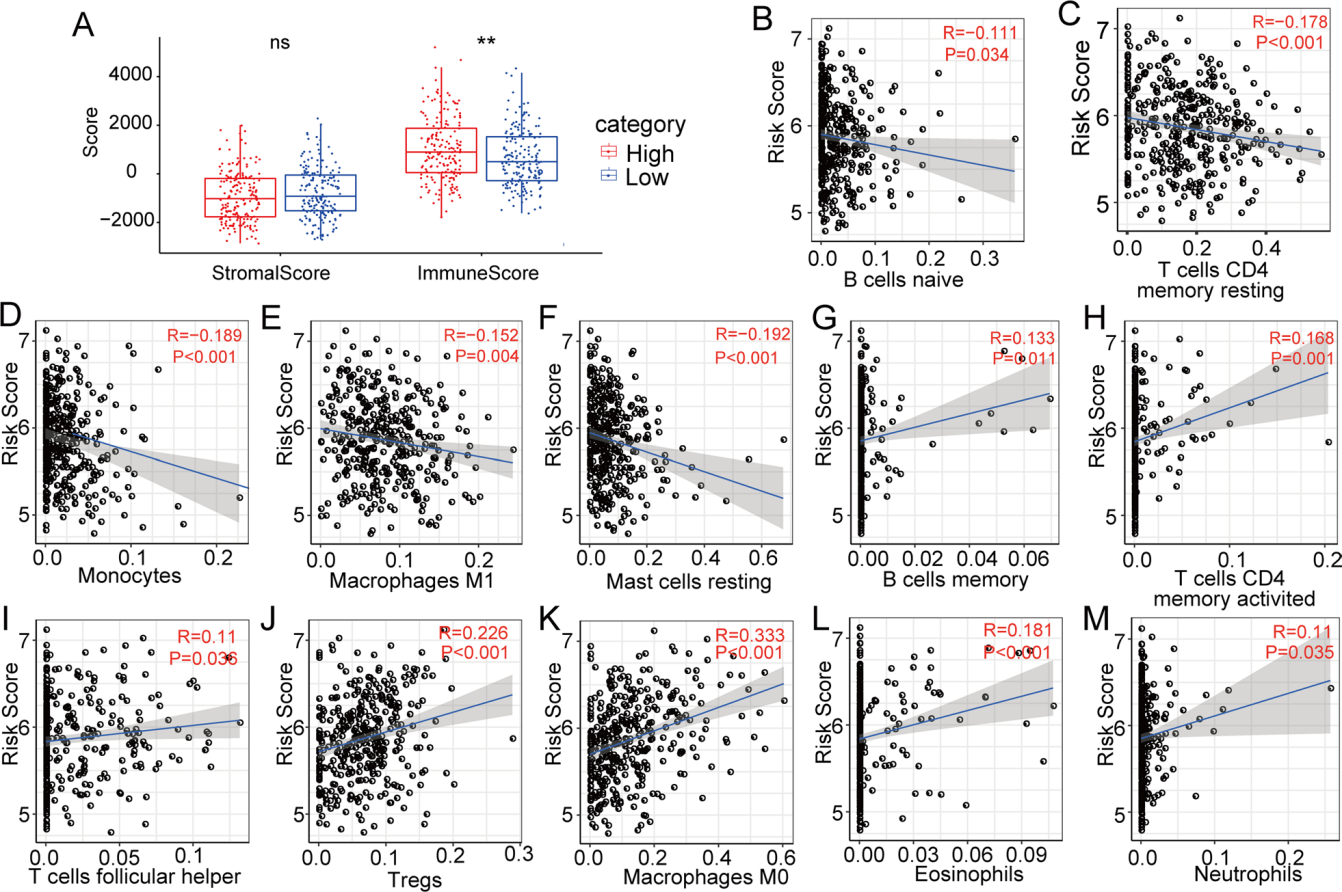
ESTIMATE was performed to calculate the immune and matrix scores for each patient. **a** Comparison of the immune score and matrix score in high- and low-risk groups. **b–m** Correlation analysis of immune cell abundance and risk score

To estimate the effect of the 3-gene model on the TIME of HCC, we analysed the association between the risk score and infiltration levels of various types of immune cells by the ESTIMATE method. The results showed that the immune score was higher in the high-risk group than in the low-risk group, while the matrix scores in the high- and low-risk groups were not significantly different (Fig. 6a). Then, the correlation between the abundance of 22 immune cells and the risk score was calculated by Pearson’s correlation analysis. We found that the risk score was negatively correlated with the abundances of naïve B cells, CD4 memory resting T cells, monocytes, M1 macrophages, and resting mast cells (Fig. 6b–f). The risk score was positively correlated with the abundances of memory B cells, activated CD4 T cells, follicular helper T cells, Tregs, M0 macrophages, eosinophils and neutrophils (Fig. 6g–m). Based on the above results, we speculated that this risk model is involved in immune microenvironment regulation and might affect the intratumoural antitumour immune response.

### Immunotherapy predictive efficacy of the 3-gene model

**Fig. 7:**
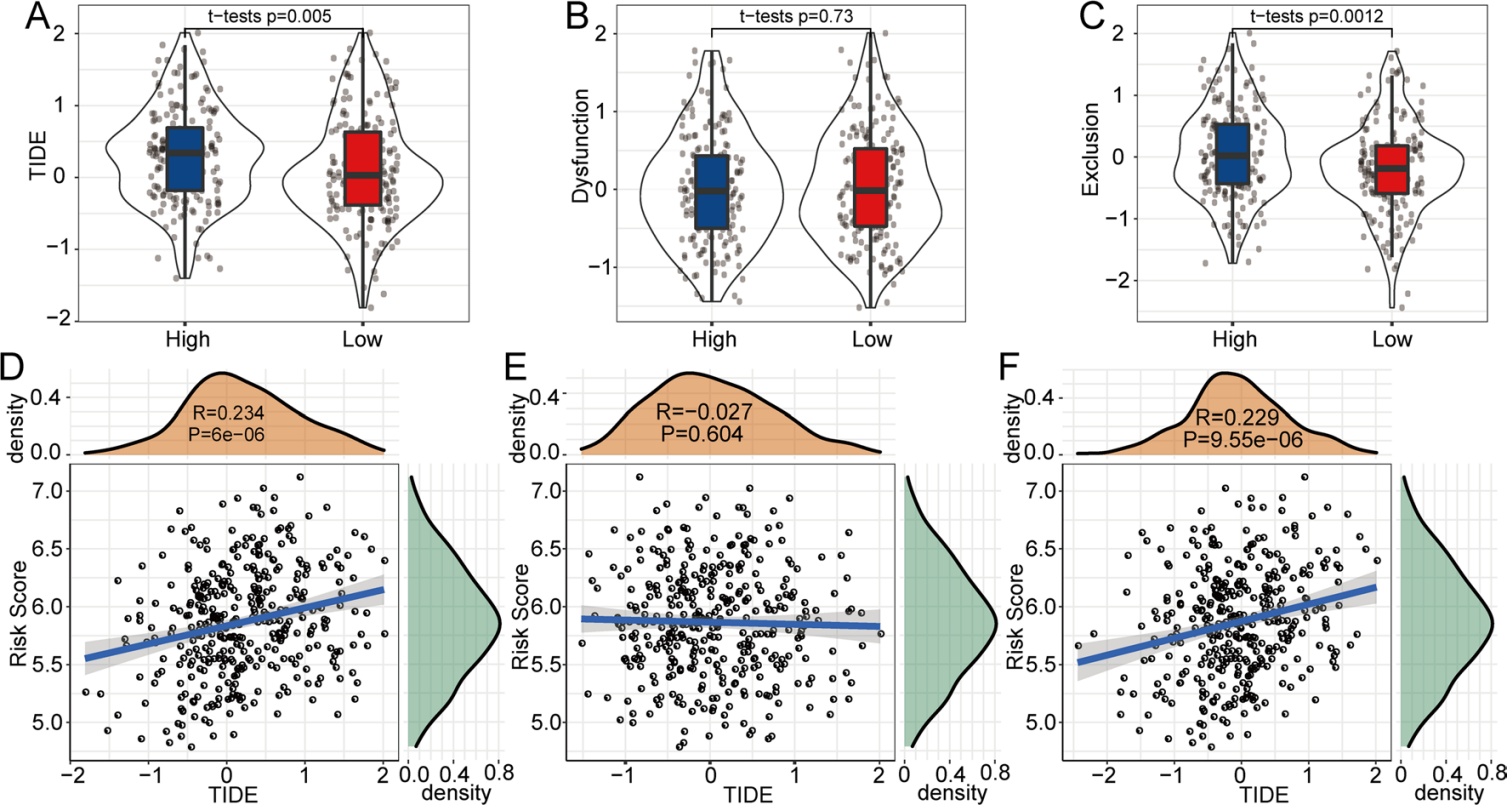
Analysis of the difference in TIDE scores between high- and low-risk groups. **a** In TCGA-LIHC, the TIDE score was low in the low-risk group. **b** T cell dysfunction scores were not significantly different in the high- and low-risk groups. **c** The high-risk group had a higher T cell exclusion score than the low-risk group. **d–f** Correlation analysis between the risk score and the TIDE score, T cell dysfunction score and T cell exclusion score. Abbreviations: TIDE, Tumour immune dysfunction and exclusion; TCGA-LIHC, The Cancer Genome Atlas Liver Hepatocellular Carcinoma

Here, we used TIDE software to evaluate the response to immunotherapy of patients in high- and low-risk groups. A higher TIDE prediction score represented a higher possibility of immune escape, indicating that the patients were less likely to benefit from immunotherapy. In TCGA-LIHC, the TIDE score of the low-risk group was significantly lower than that of the high-risk group (Fig. 7a). Furthermore, we discovered that T cell dysfunction scores were not significantly different in the high- and low-risk groups (Fig. 7b). The high-risk group had a higher T cell exclusion score than the low-risk group (Fig. 7c). Furthermore, the results of the correlation analysis showed that the risk score was markedly correlated with the TIDE score and T cell exclusion score (Fig. 7d–f). Taken together, the evidence might demonstrate why patients with high risk scores have a poor prognosis and why patients with high risk often exhibit a poor response to immunotherapy.

### Clinical characteristics associated with the 3-gene signature in HCC

**Fig. 8:**
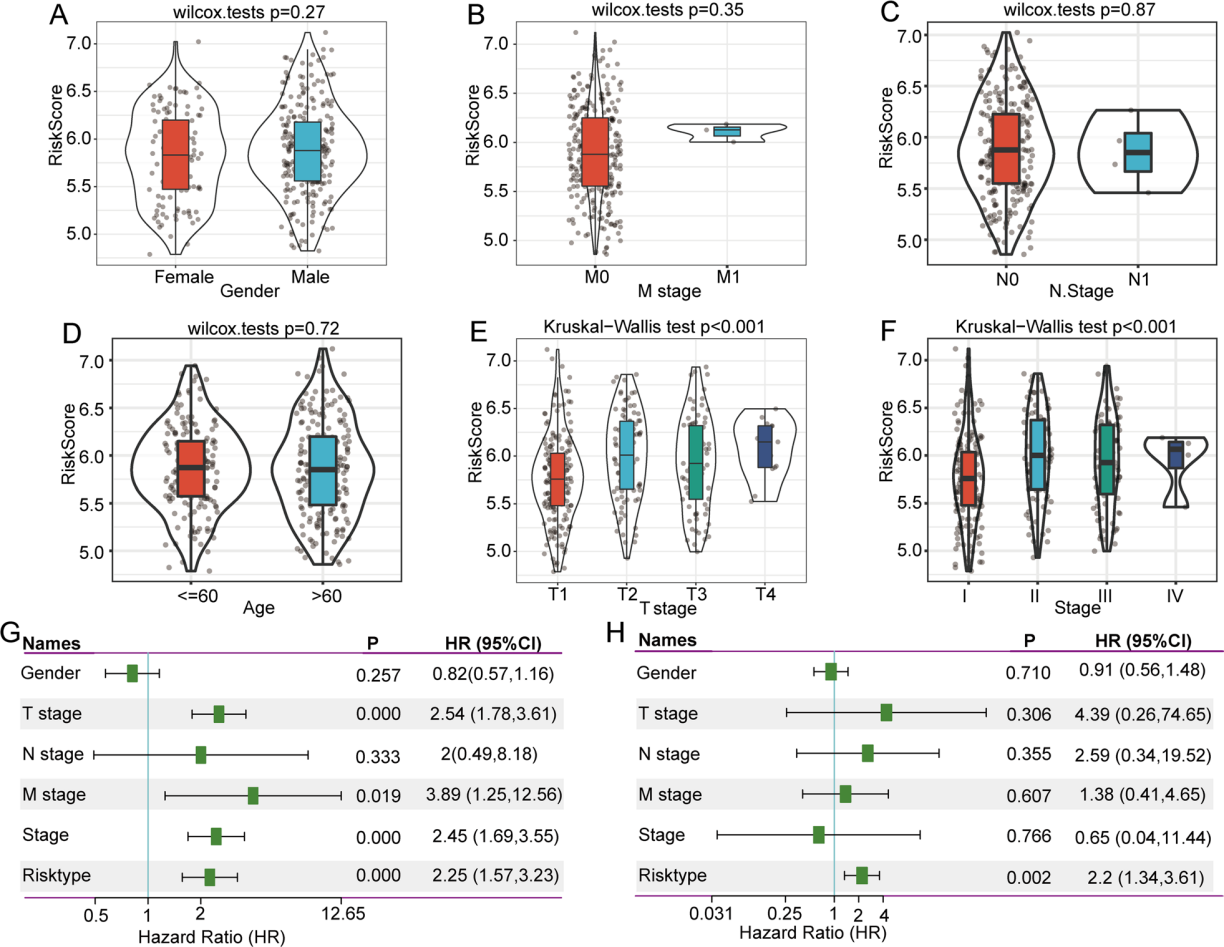
Clinical application of the 3-gene signature in HCC. The difference in risk score by sex (**a**), M stage (**b**), N stage (**c**), age (**d**), T stage (**e**) and stage (**f**) of HCC. Univariate (**g**) and multivariate (**h**) Cox regression analysis in TCGA-LIHC. Abbreviations: TCGA-LIHC, The Cancer Genome Atlas Liver Hepatocellular Carcinoma; HCC, hepatocellular carcinoma

After confirming the performance of the 3-gene signature in predicting the response to immunotherapy of patients with HCC, we subsequently investigated the association between clinical characteristics and the risk score. Although the differences in risk score by sex, M stage, N stage, and age were not statistically significant (Fig. 8a–d), the risk score was significantly different among tumour and T stages, and the risk score was higher in more advanced HCC (Fig. 8e and f).

To further explore the clinical application of the 3-gene model in predicting the prognosis of patients. We utilized univariate and multivariate Cox regression analyses in TCGA-LIHC. The results showed that the risk score was significantly correlated with prognosis (Fig. 8g). In addition, multivariate Cox regression analysis further confirmed that the risk score was an independent risk factor for HCC (Fig. 8h). Collectively, these results confirm that the 3-gene signature has excellent prognostic efficiency.

## Discussion

scRNA-seq has emerged as a useful tool for transcriptional classification of cell types in various cancers. Here, we performed HCC scRNA-seq data from the GEO database to define the cell subpopulations in tumours, and we found multiple subgroups of 5 cell types, including liver bud hepatic cells, CD4 + cytotoxic T cells, dendritic cells, Kupffer cells, and liver progenitor cells. Specifically expressed gene markers might serve as specific markers to identify cell subgroups in a large set of samples. In addition, we screened the key genes related to immune cell subsets in HCC and constructed a three-gene risk model that had excellent prognostic efficiency and might serve as a biomarker for immunotherapy response. Similarly, Liang et al. [22] used scRNA-seq to analyse the heterogeneity of tumour immune cells and established a risk model for predicting the prognosis of ovarian cancer patients. Zheng et al. [17] screened six hub genes related to prognosis from GEO oesophageal squamous cell carcinoma (ESCC) datasets and TCGA ESCC datasets, and the results of scRNA-seq showed that the expression of hub genes was significantly higher in normal tissues and cells. Further Kaplan–Meier survival analysis and immune infiltration analysis indicated that the hub genes were promising biomarkers for ESCC diagnosis and prognosis [17]. scRNA-seq was also adopted to decipher the cell-to cell interactions inside gliomas, and the identified autocrine ligand-receptor signal pairs were found to significantly affect the prognosis of glioma patients [25]. Taken together, the findings indicate that scRNA-seq technology could help to effectively dissect the TIME and identify potential prognostic biomarkers.

Here, we performed differential analysis on gene expression data from the TCGA database. Three upregulated DEGs (cystatin B (CSTB)**,** transaldolase 1 (TALDO1) and clathrin light chain A (CLTA)**)** that belonged to monocyte (C21) marker genes might be applied as potential biomarkers for immunotherapy. CSTB, a member of the cystatin superfamily, is an inhibitor of cysteine proteases. Dysregulated expression of CSTBA has been reported to be involved in various cancers. For example, the expression of CSTBA was increased in serum and might be an early-stage diagnostic biomarker for HCC [26] and ovarian epithelial tumours [27]. CSTB has also been reported to serve as a prognostic biomarker for bladder cancer [28], lung cancer and colorectal cancer [29, 30]. Wu et al. reported that the expression of TALDO1 was increased in upper tract urothelial carcinoma tissues and that upregulated TALDO1 expression was correlated with large tumour size, advanced stage, and distant metastases [31]. In addition, genetic polymorphisms in TALDO1 were closely correlated with squamous cell carcinoma of the head and neck [32]. A better understanding of the molecular mechanisms of the 3-gene model in HCC pathogenesis to validate its clinical applications is needed for the further development of novel diagnostic and prognostic biomarkers.

In this work, we jointly analysed scRNA-seq data and the gene expression profile of bulk RNA-seq data. The results both improve our understanding of the heterogeneity of the TIME at the single-cell level and provide a 3-gene model based on prognosis-related genes. Additionally, the research strategies used in this study might also be suitable for other cancers. However, there were several limitations in this study. First, the size of sample was relatively small. Second, the functional experiments and underling molecular mechanism of the 3 genes are needed. Third, the model was generated with HCC tissues, which cannot diagnose tumour at the early stage. In future studies, we plan to detect the expression of the three genes in circulating immune cells, which might contribute to increasing the early diagnosis rate for HCC.

## Conclusion

By integration of bulk RNA-seq and scRNA-seq, we analysed the heterogeneity of the TIME at the single-cell level, and we constructed a 3-gene model that could accurately evaluate the survival outcome and immunotherapy response of patients with HCC.

## Supplementary Information

**Additional file 1: Table S1.** The information of primers sequences for qRT-PCR assay.

**Additional file 2: Figure S1.** Quality control charts for each sample before and after single-cell data filtering.

**Additional file 3: Figure S2.** The flow chart of the analysis procedure in our study.

**Additional file 4: Figure S3.** Single-cell data clustering dimensionality reduction analysis. (a–b) PCA dimension reduction analysis. (c) Expression of the top 5 marker genes in 25 clusters.

**Additional file 5: Figure S4.** Functional enrichment analysis of genes in the brown module. (a) BP annotation map of genes in the brown module. (b) MF annotation map of genes in the brown module. (c) CC annotation map of genes in the brown module. (d) KEGG annotation diagram of brown module genes. Abbreviations: MF, molecular function; BP, biological process; CC, cellular component; KEGG, Kyoto Encyclopedia of Genes and Genomes.

## Data Availability

The datasets used and/or analysed during the current study are available from the corresponding author upon reasonable request.

## Abbreviations

HCC: Hepatocellular carcinoma
scRNA-seq: Single-cell RNA sequencing
GEO: Gene Expression Omnibus
TCGA: The Cancer Genome Atlas
ICGC: International Cancer Genome Consortium
CIBERSORT: Estimating relative subsets of RNA transcripts
WGCNA: Weighted gene coexpression network analysis
LASSO: Least absolute shrinkage and selection operator
TIME: Tumour immune microenvironment
RNA-seq: RNA sequencing
PT: Primary tumour
PVTT: Portal vein tumour thrombus
MLN: Metastatic lymph node
NLT: Normal liver tissue
PCA: Principal component analysis
TIDE: Tumour immune dysfunction and exclusion
qRT-PCR: Quantitative real-time PCR
BP: Biological process
CSTB: Cystatin B
TALDO1: Transaldolase 1
CLTA: Clathrin light chain A
