# Supplementary material for: A novel prognostic model based on single-cell RNA sequencing data for hepatocellular carcinoma
Source: Cancer Cell Int. 2022 Jan 25;22:38. doi: 10.1186/s12935-022-02469-2 (PMC8787928; doi:10.1186/s12935-022-02469-2)
Supplement: Supplementary file 1 — Additional file 1: Table S1. The information of primers sequences for qRT-PCR assay. [file 12935_2022_2469_MOESM1_ESM.docx]

Table S1. The information of primers sequences for qRT-PCR assay.

| Primer name | Sequence (5'-3') |
| --- | --- |
| CLTA-F | CGGAAGCAAGAAGCAGAGTG |
| CLTA-R | TGTTGTTTGCTTTTGTTTTCTGTAG |
| TALDO1-F | GCATCCACTGCAACATGACG |
| TALDO1-R | TGCCACATGCCAATCAAGGA |
| CSTB-F | GCCGTGTCATTCAAGAGCCA |
| CSTB-R | TGCTTGGCTTTGTTGGTCTG |
