# Supplementary figures and images for: A novel prognostic model based on single-cell RNA sequencing data for hepatocellular carcinoma
Source: Cancer Cell Int. 2022 Jan 25;22:38. doi: 10.1186/s12935-022-02469-2 (PMC8787928; doi:10.1186/s12935-022-02469-2)

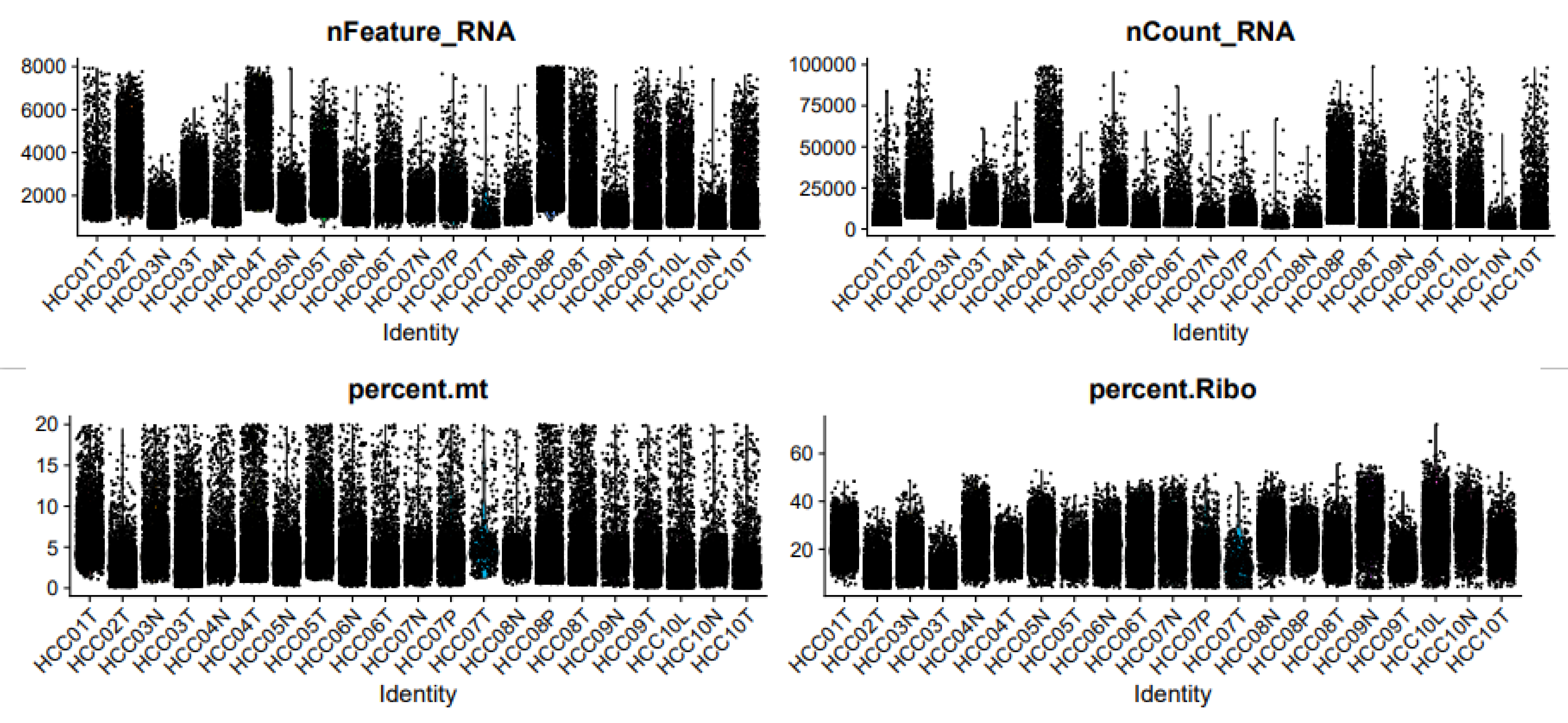

Supplement: Supplementary file 2 — Additional file 2: Figure S1. Quality control charts for each sample before and after single-cell data filtering. [file 12935_2022_2469_MOESM2_ESM.tif]

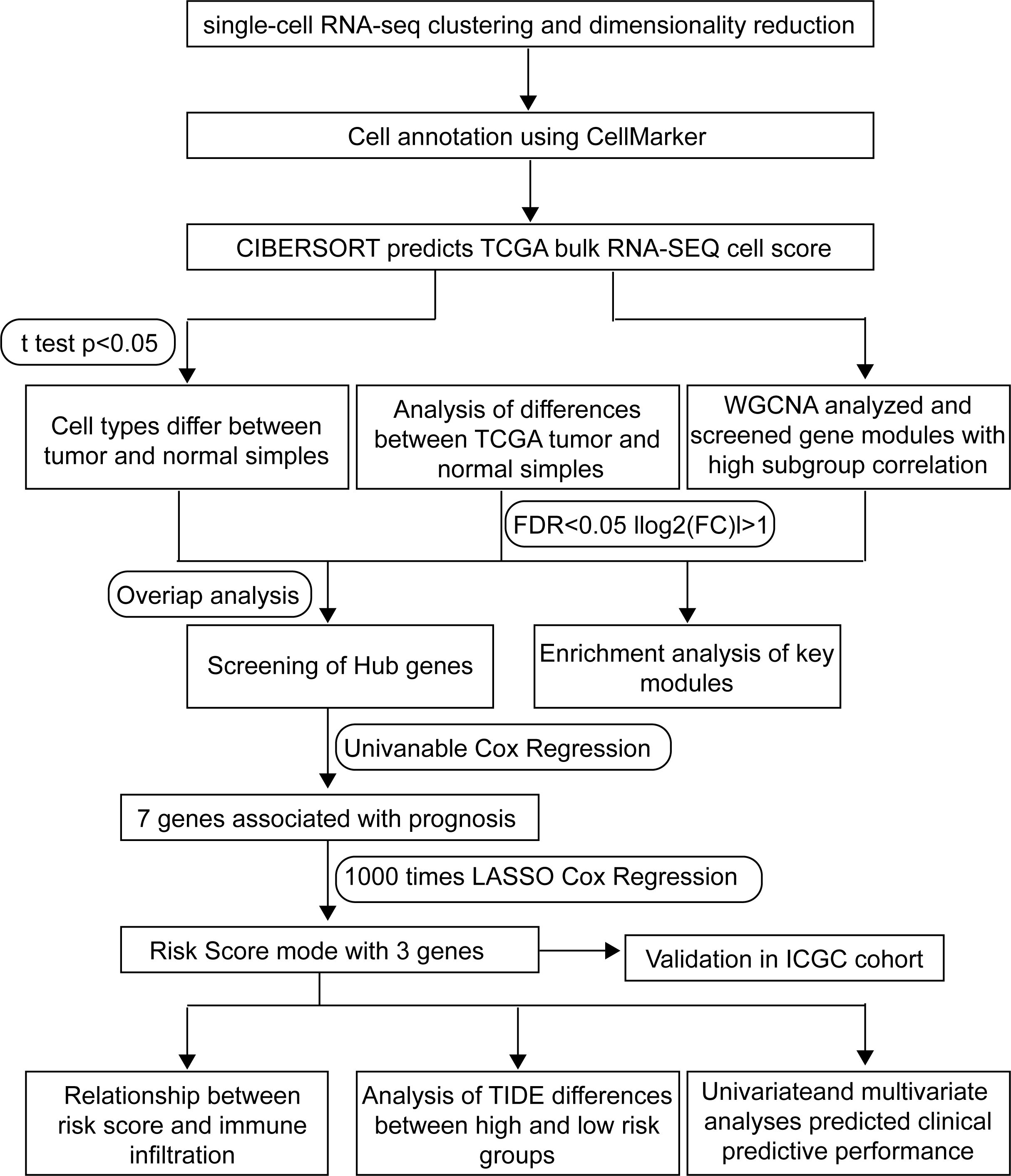

Supplement: Supplementary file 3 — Additional file 3: Figure S2. The flow chart of the analysis procedure in our study. [file 12935_2022_2469_MOESM3_ESM.tif]

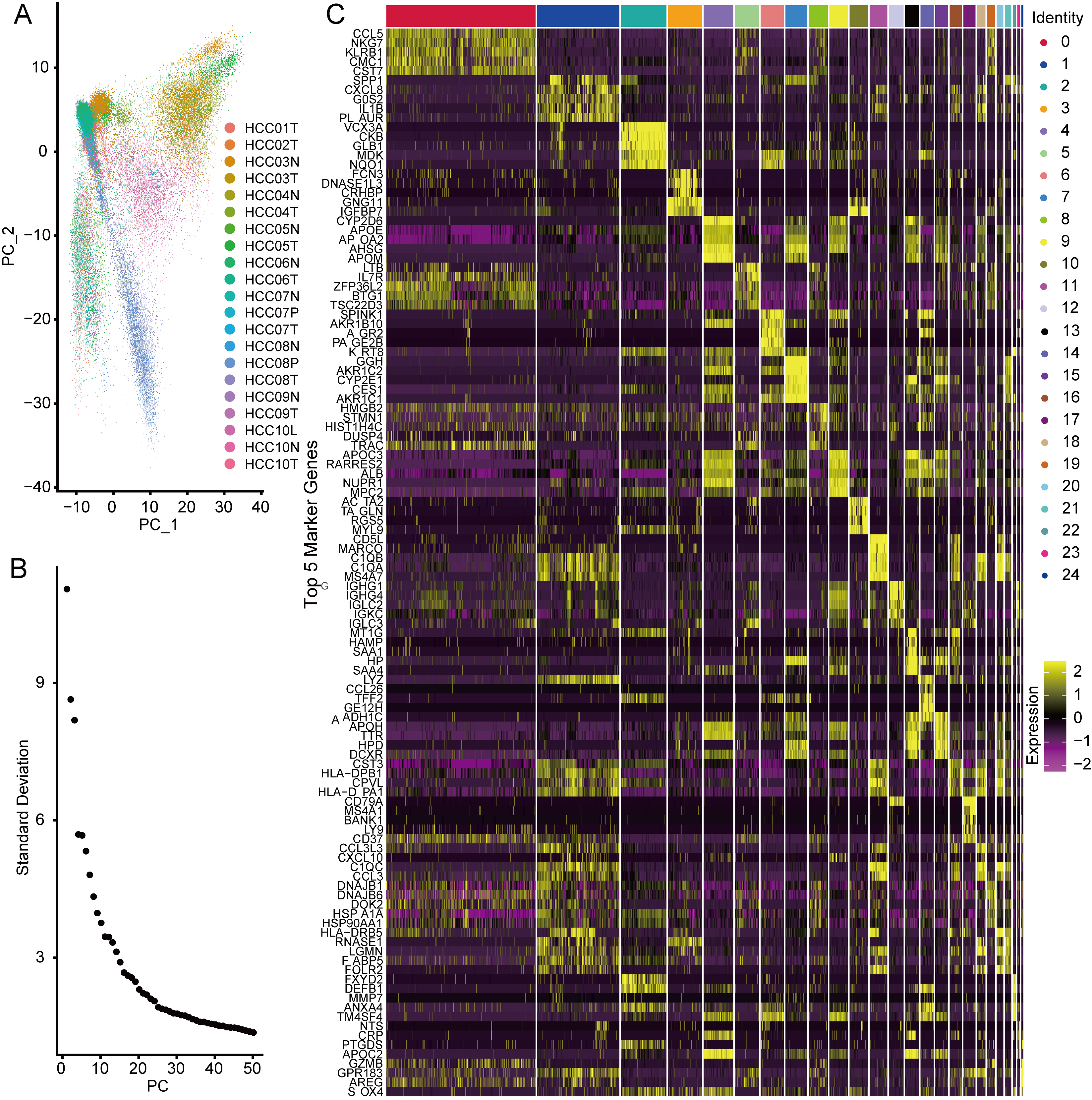

Supplement: Supplementary file 4 — Additional file 4: Figure S3. Single-cell data clustering dimensionality reduction analysis. (a–b) PCA dimension reduction analysis. (c) Expression of the top 5 marker genes in 25 clusters. [file 12935_2022_2469_MOESM4_ESM.tif]

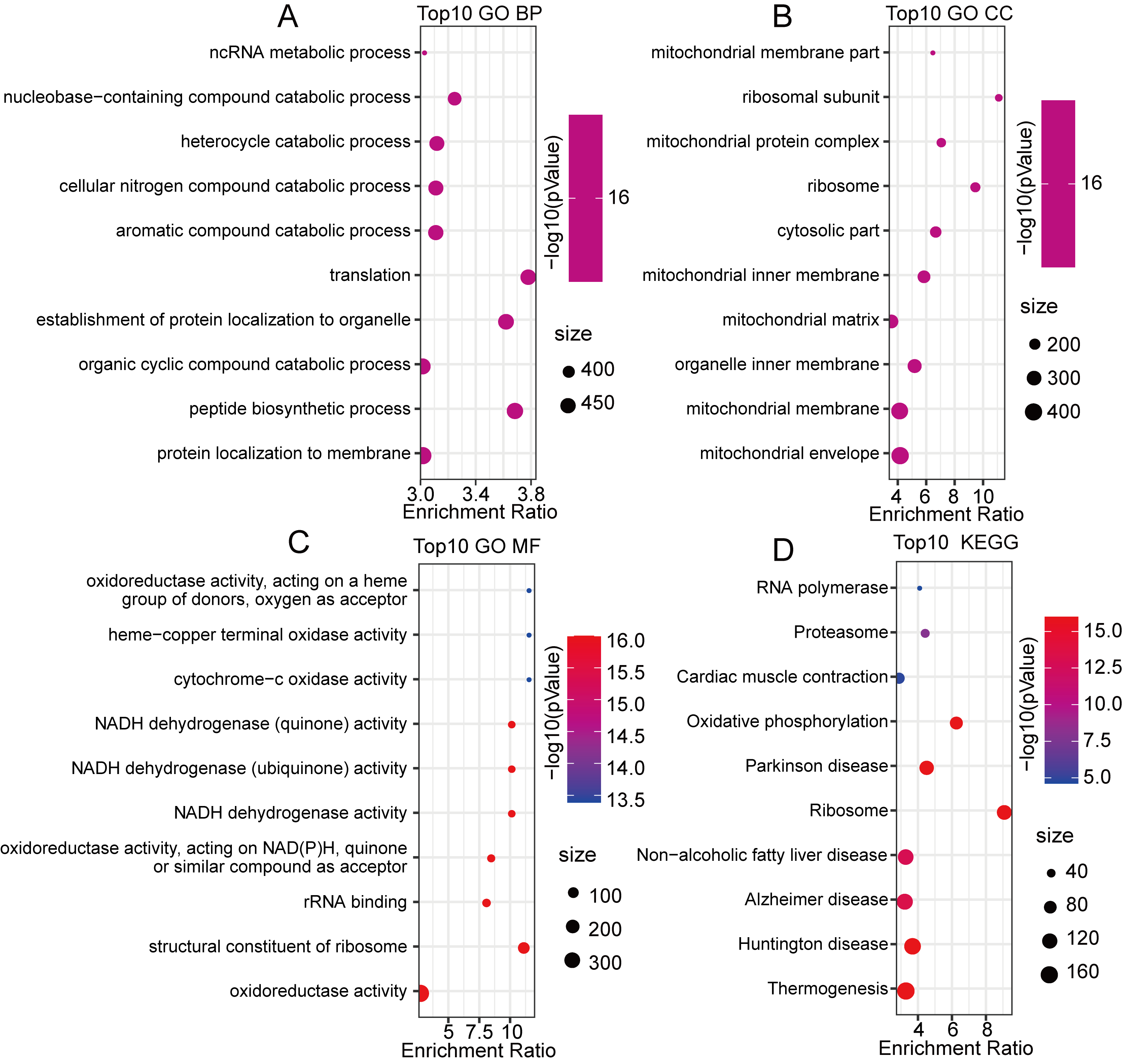

Supplement: Supplementary file 5 — Additional file 5: Figure S4. Functional enrichment analysis of genes in the brown module. (a) BP annotation map of genes in the brown module. (b) MF annotation map of genes in the brown module. (c) CC annotation map of genes in the brown module. (d) KEGG annotation diagram of brown module genes. Abbreviations: MF, molecular function; BP, biological process; CC, cellular component; KEGG, Kyoto Encyclopedia of Genes and Genomes. [file 12935_2022_2469_MOESM5_ESM.tif]
